# Fold combinations in multi-domain proteins

**DOI:** 10.6026/97320630015342

**Published:** 2019-05-15

**Authors:** Nagarajan Naveenkumar, Gayatri Kumar, Ramanathan Sowdhamini, Narayanaswamy Srinivasan, Sneha Vishwanath

**Affiliations:** 1 National Center for Biological Science, GKVK Campus, Bengaluru, Karnataka, India - 560065; 2 Bharathidasan University, Tiruchirappalli, Tamil Nadu, 620024, India; 3 Molecular Biophysics Unit, Indian Institute of Science, Bengaluru, Karnataka, India - 560012

**Keywords:** domain architecture, domain folds, multi-domain proteins, protein evolution, protein structure

## Abstract

Domain-domain interactions in multi-domain proteins play an important role in the combined function of individual domains for the overall biological activity of the protein. The functions of the tethered domains are often coupled and hence, limited numbers of domain architectures with defined folds are known in nature. Therefore, it is of interest to document the available fold-fold combinations and their preference in multi-domain proteins. Hence, we analyzed all multi-domain proteins with known structures in the protein databank and observed that only about 860 fold-fold combinations are present among them. Analyses of multi-domain proteins represented in sequence database result in recognition of 29,860 fold-fold combinations and it accounts for only 2.8% of the theoretically possible 1,036,080 (1439C2) fold-fold combinations. The observed preference for fold-fold combinations in multi-domain proteins is interesting in the context of multiple functions through structural adaptation by gene fusion.

## Background

Domains are the structural, functional, and evolutionary units of proteins. Majority of the proteins encoded in genomes of all the taxa consists of multiple domains 1, 2. Tethering of domains in multi-domain proteins confers folding, functional and stability advantages. For example, the folding rates of many multi-domain proteins are reported to be faster than the homologous single domain proteins 3, the stability of the multi-domain proteins is known to be better than the homologous single-domain proteins for many cases 4 and domain-domain interfaces are observed to play an important role in allosteric regulation of proteins 5-7. Multidomain proteins have evolved from single domain proteins through many duplication and adaptive events 3. These events have led to the emergence of various unique and novel functions using an existing repertoire of domains 8-11. Function of multiproteins is influenced by architecture of domain arrangement and the interaction between domains 12, 13. Different domain combinations in proteins bring about the functional diversity in proteins 14. A limited repertoire of domain combinations in proteins has been observed 15, 16, with some domain combination being more frequent. Limited number of domain combinations in proteins suggests that tethering of domains in multi-domains proteins is not a chance event in evolution 1, 13, 17. The combination of domains in multi-domain proteins is a driven process during evolution that optimises the function of proteins 15, 17. Here we studied the repertoire of domain folds occurring in multi-domain proteins and, more importantly, the preferred combinations of folds in multi-domain proteins. With this aim, we analysed all the multi-domain proteins of known 3-D structure available in the Protein Databank (PDB) 18, 19. Information on classification of a protein domain fold has been obtained from the SCOP database 20, 21. From the dataset of multi-domain proteins with known structure, we observe only 35% of the known folds in multi-domain proteins and 860 fold-fold combinations out of 1,036,080 theoretical possibilities. Further, integration of the sequence data in the dataset increases the number of folds observed in multi-domain protein to 1021 (71% of the known folds) and the number of fold-fold combinations to 29860. Although, ~35 times increase is observed in the number of fold-fold combination on integrating the sequence data, the number is far less than the number of theoretically possible fold-fold combinations (1,036,080 for 1439 folds). The analysis shows that only a few (~2.8%) of the fold-fold combinations are observed in multi-domain proteins. This limitation could be due to geometrical constraints associated with fold-fold associations in multi-domain proteins. Our analysis also highlights the incompleteness of representation of multi-domain proteins in the current version of PDB, as only 504 folds out of 1439 folds described in SCOPe are observed.

## Methodology

### Dataset of multi-domain proteins from SCOPe � structure dataset:

We retrieved 3-D structures of proteins solved by X-ray diffraction method or Nuclear Magnetic Resonance (NMR) from the Protein Data Bank (PDB) 18. All the PDB entries with no SCOP annotations available in SCOPe 22 were discarded. 244,326 protein domains in SCOP ver. 2.07 were filtered for genetic domains, discontinuous domain and His-tag domain (SCOPe code: l.1.1.1). The resulting 217,523 protein domains were filtered for single-domain entries. 71,016 proteins domains were observed to be part of multi-domain proteins. Fold-fold combinations in these multi-domain proteins were enumerated only if the domains are interacting. Domain-domain interactions were defined between the domains in multi-domain proteins if at least five residue-residue interactions are observed between domains. The interactions between domains were identified using Protein Interaction Calculator (PIC) 23. For the analyses here, we have not taken into consideration the frequency of observation of a given fold-fold combination; we only consider if the association between two specific folds is observed or not.

### Dataset of multi-domain proteins from Pfam � sequence dataset:

Domain architecture was retrieved from Pfam database 24. We generated corresponding SCOP domain architectures using the previously described protocol by Kumar et al. 25 for each domain architectures in Pfam. The method described by Kumar et al. allows mapping of Pfam domain to SCOP domain. We extended the annotation from individual Pfam domain to SCOP domain mapping to domain architecture. The Pfam ver. 31 and SCOPe 2.07 version were used for the analysis. For the fold-fold combination enumeration, it is assumed that all the domains in a multi-domain protein interact with one another. Although, it is likely to add false positives, it gives an upper limit to the number of fold-fold combinations observed in nature.

## Results

### Limited set of folds and fold-fold combinations:

**Figure 1:**
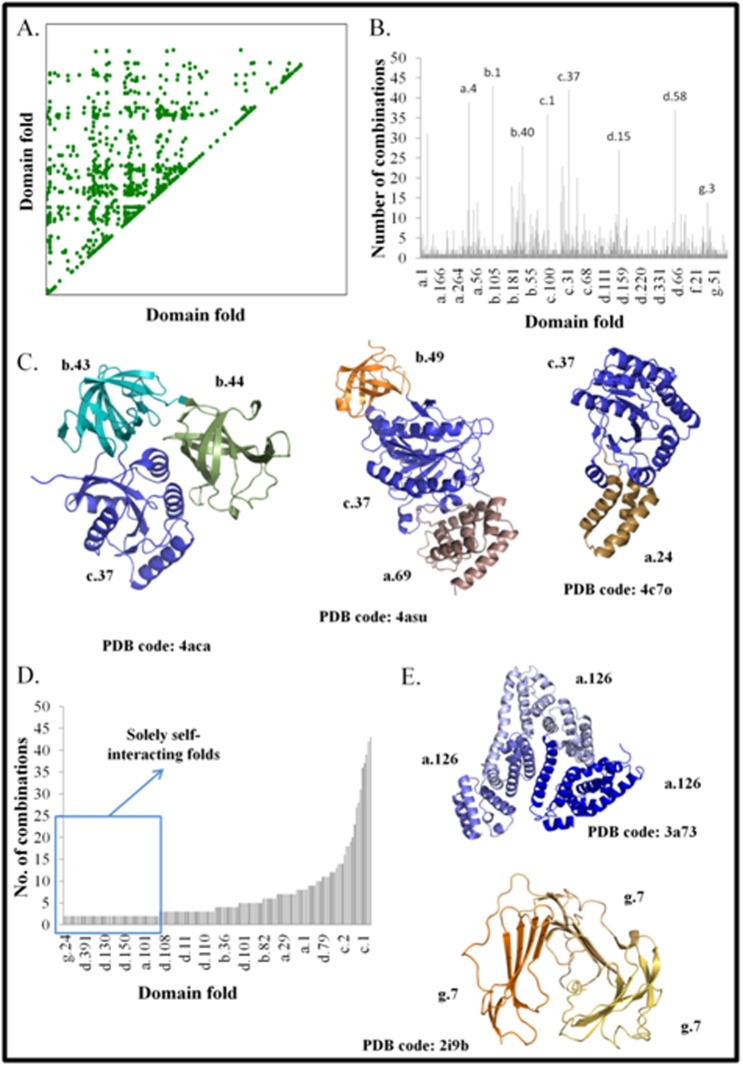
Fold-fold combinations and their features. A. Dot matrix of the fold-fold combinations observed in the dataset of known 3-D structures. The X-axis and the Y-axis represent the 1439 folds defined in SCOPe 29 database. A dot in the matrix indicates the observation of association between the corresponding domain folds. B. Distribution of the number of combinations observed for the domain folds in multi-domain proteins. SCOP codes of few of the fold with high number of combinations are indicated above the bar. The X-axis represents the folds of the domain and the Y-axis represents the number of combinations observed. C. Representative examples of a fold - nucleoside triphosphate hydrolases fold (c.37)interacting with different domains (PDB code: 4aca 30, 4asu 31 and 4c7o 32). The c.37 fold is represented in blue. D. The distribution of the number of combinations of self-interacting folds. The folds, which are 'solely self-interacting', are boxed in blue. The X-axis represents the domain fold and the Y-axis represents the number of combinations. E. Representative example of solely selfinteracting folds: serum albumin-like fold (a.126) and snake toxinlike fold (g.7) (PDB codes: 3a73 33 and 2i9b 34 respectively)

There are 1439 folds reported in SCOPe version 2.07. In the dataset of multi-domain proteins of known structure, only 504 folds are observed for constituent domains. On enumerating the fold-fold preferences in multi-domain proteins, only 860 fold-fold combinations are observed out of 1,036,080 theoretical combinations calculated for 1439 folds (Figure 1A). The observed fold-fold combinations are listed in Table 1. On further analysis of the number of fold-fold combinations observed for each of the 504 folds, it is observed that 430 (~79%) folds interact with fewer than five folds and the rest ~21% interacts with more than five folds (Figure 1B). Few examples of folds with number of combinations greater than five are - DNA/RNA-binding 3-helical bundle fold (a.4), immunoglobin-like beta sandwich fold (b.1), OB-fold (b.40), TIM beta/alpha-barrel fold (c.1), P-loop containing nucleoside triphosphate hydrolases fold (c.37), beta-Grasp fold (ubiquitinlike)( d.15), Ferredoxin-like fold (d.58) and Knottins fold (g.3). Three of the fold combinations of c.37 fold are shown in Figure 1C. The total number of combinations observed for c.37 fold is 42.

Interestingly, domains corresponding to ~28% (155 folds out of 504 folds) of the folds interact with domains of same fold (dots along the diagonal in Figure 1A). Out of the 34,002 domain-domain interactions observed, 10,591 domain-domain interactions are observed between same fold. Interestingly, about 2,697 domaindomain interactions between same folds are not domain-repeats (i.e. the domains belonged to different families). The occurrence of such domain-domain interactions between same folds but from different superfamilies supports the concept of evolution of these domains from gene-duplication events. Interestingly, 48 of these self-interacting folds interact with only domains of same fold (boxed in blue in Figure 1D). Such folds henceforth will be referred as 'solely self-interacting folds'. Examples of solely self-interacting folds are serum albumin-like fold (a.126) and snake toxin-like fold (g.7) (Figure 1E). It should be noted that observation of solely selfinteracting folds is not due to limited number of occurrence of these folds in SCOPe database 22 (Table 2). For example, a.126 and g.7 are represented 488 and 269 times respectively in the SCOPe database. These observations imply that certain folds prefer to tether with a domain of same fold. The geometrical compatibility between the folds could be a defining feature of folding of proteins having interacting domains of same folds. Many of the folds that interact with domains of same fold are observed to interact with many other folds (extreme right column bars in Figure 1D) as well. This observation implies that certain folds have geometrical features that are compatible for interaction with many different folds. One of the many reasons for this could be the number of functions that are associated with such folds and are discussed in detail in the following section.

### Fold-fold combinations and associated functions:

We investigated potential biological implication of substantial proportion of domain folds (~21%) preferring to interact with many domain folds. For this, we used the functional annotations from SUPERFAMILY database 26 for the superfamilies listed under each of these domain folds 11, 27. The functional annotation of each superfamily was extrapolated to the fold i.e. each fold is assigned all the functions that each of its constituent superfamily has been assigned with. In the SUPERFAMILY database, the function associated with majority of the family members is assigned to the superfamily. It has to be noted that functions definition used in SUPERFAMILY database is with respect to the most common role of the domain in proteins, in a particular pathway or in the cell/organism. The definition is mix of the definition of 'biological process' and 'molecular function' used in the Gene Ontology annotation 28. It has to be noted that the information of the function of the folds could be retrieved only for 470 folds out of 504 folds.

**Figure 2:**
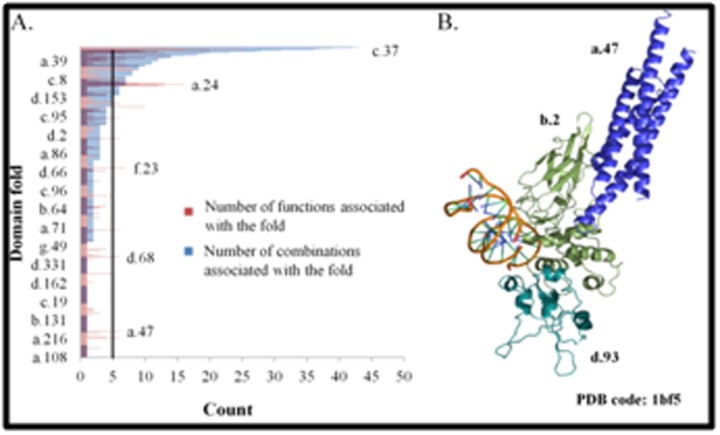
Functional annotations of domain folds. A. Bar plot representation of the number of the combinations associated with folds and the number of functions associated with folds. A vertical line is drawn at five. The X-axis represents the count of the number of fold combinations with a given fold (coloured as blue bars) and the number of functions (coloured as red bars) associated with a fold and the Y-axis represents the domain folds. B. An example of protein (PDB code: 1bf5 35) with fold-fold combination of STATlike fold (a.47) and common fold of diphtheria toxin/transcription factors/cytochrome f fold (b.2). The fold a.27 is coloured in blue and the fold b.2 is coloured in green

Majority of the folds follow the trend of higher the number of functions associated with a fold, higher is the number of fold combinations (Figure 2A).Folds that have 20 or more number of fold-fold combinations and the number of functions associated with the folds are listed in Table 3. Few examples that do not follow this trend are also observed i.e. folds, which have fewer than 5 fold-fold combinations are associated with substantial number of functions. For example, STAT-like fold (a.47) has only one fold-fold combination i.e. with common fold of diphtheria toxin/transcription factors/cytochrome f (b.2) but six associated functions (Figure 2B). The two folds share 4 out of 6 functions, suggesting that the tethered domain influences the functions of the domain folds.

### Limited repertoire of fold-fold combinations

**Figure 3:**
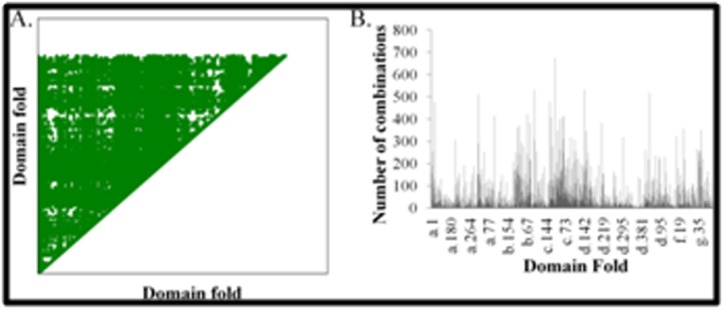
Fold-fold combinations observed in a dataset of sequences of multi-domain proteins. A. Dot matrix of the fold-fold combinations observed in the sequence dataset. The X-axis and the Y-axis represents the 1439 folds defined in SCOPe 29 database. A dot in the matrix indicates the observation of association between the corresponding domain folds. B. Distribution of the number of combinations observed for the folds represented in multi-domain proteins in the sequence dataset. The X-axis represents the folds of the domain and the Y-axis represents the number of combinations observed

The structural information available on multi-domain proteins is limited due to the technical difficulties in crystallising multidomain proteins and the size limitation on the structure elucidation by nuclear magnetic resonance (NMR). Since only a minor fraction of PDB represents multi-domain proteins (~35%) in contrary to the proportion of multi-domain proteins encoded in genomes (~75% on average), it is imperative to include sequence data, which is much larger, in the our analysis dataset. For this, we mapped the information on sequence domains available in the Pfam database (ver. 31) 24 to SCOP domains (SCOPe version 2.07 22). Information on domain architecture was retrieved from Pfam database 24. We generated the corresponding SCOP domain for each domain architecture. For the analysis of the sequence dataset, it is assumed that all the domains in a multi-domain protein interact with one another. Although, it adds a greater number of fold-fold combinations that may not be actually observed, it would give an upper limit to the number of fold-fold combinations possible in nature. 4095 of the sequence domains documented in Pfam ver. 31 could be mapped to structural/evolutionary domain documented in SCOP database using methods discussed previously by Kumar et al. 25. Mappings where Pfam domain corresponded to two different SCOP folds were discarded. 374,319 multi-domain Pfam architectures were mapped to 29,860 SCOP fold-fold combinations (Figure 3A). These fold-fold combinations represented 1021 folds out of 1439 folds in SCOPe database. Although, the number of fold-fold combinations deduced from the sequence database is ~35 times more than the fold-fold combinations observed in the structure dataset, the number is still far less than the number of theoretically possible fold-fold combinations (~1036080 fold-fold combinations for 1439 folds). These observations suggests strongly that only a few (~2.8%) of the fold-fold combinations are preferred in multi-domain proteins. Thus, only few fold-fold combinations are selected by nature during evolution of multi-domain proteins. The selection of the fold-fold combinations could be because of geometrical constraints during evolution or/and the functional constraints, like coupling of functions, allostery regulation etc., on the constituent domains of multi-domain proteins. 202 folds in the sequence dataset have fold-fold combinations fewer than five (Figure 3B), with 31 folds being in common between sequence and structure dataset. Since ~35 times more fold-fold combinations are observed in the sequence dataset than the structure dataset, it stresses on the need of structure elucidation of multi-domain proteins to understand better the structure and functions of multi-domain proteins.

## Discussion:

**Figure 4:**
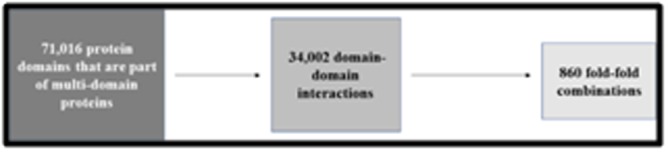
Flowchart representing the size of data considered for enumeration of fold-fold combination for the PDB dataset.

Majority of the proteins encoded in genomes of all the life forms have more than one domain. The presence of multiple domains in a protein confers stability (e.g. by forming stabilising interactions between domains 4), functional (e.g. allostery regulation of domains by tethered domains 5-7), and folding (e.g. prevention of aggregation during folding by independent folding of the domains 3) advantages. Because of these advantages of tethering of domain, few of the domain combinations always occur together, such combinations of domains are known as supra-domains 27. Since domain-domain interactions have been reported to be important for many of the functions of the proteins 5-7 and it is one of the reasons for selection of certain domain-domain combinations during evolution, here we addressed the question whether the geometrical compatibility between domains to interact restricts the number of fold-fold combinations observed in multidomain proteins. In addition, we asked whether certain folds are pre-disposed to occur in multi-domain proteins. For this, we first analysed all the available multi-domain proteins with known structure in the light of annotated domain folds. Interestingly, only 504 folds out of 1439 folds are observed in multi-domain proteins with known structures and these 504 folds form 860 fold-fold combinations (Figure 4). Repetition of analysis but using a much larger sequence data resulted in observation of 29,860 fold-fold combinations out of 1,036,080 theoretical fold-fold combinations possible for 1439 folds. 1021 folds were observed as part of multidomain protein in the sequence dataset. These observations strongly suggest that certain folds are pre-disposed to occur in multi-domain proteins and only few fold-fold combinations are selected for during evolution. The selection pressure for these foldfold combinations could be the geometrical constraints during the folding of proteins or/and the functional constraints on tethered domains to optimise the function/fitness cost of the protein during evolution. Observation of only 504 folds in the multi-domain proteins with known structures reflect the paucity of multi-domain proteins in the structure databases like PDB and stresses on the need of rigorous structural elucidation of multi-domain proteins. 21% of the folds are observed to form more than 5 fold-fold combinations. On detailed analyses, it is observed that these folds have multiple distinct functions associated with them. Another interesting feature is that few folds, which have less than 5 fold-fold combinations, have multiple distinct features associated with them. Detailed analyses showed that such domain have overlapping functions with the tethered domain. Such overlapping of functions of tethered domain again stresses the importance of domaindomain interaction in the function of proteins.

## Conclusion

The results discussed here suggest that a limited set of fold-fold combinations have been selected for multi-domain proteins during evolution. Our analyses also highlight the disparity in the number of multi-domain proteins for which structure has been elucidated and the number of multi-domain proteins encoded in the genomes of all life forms.

## Conflict of Interest

Authors declare no conflict of interest.

## Figures and Tables

**Table 1:** Fold-fold combinations observed in the structural dataset of multi-domain proteins

List of folds
a.100-a.100	a.126-a.126	a.215-a.216	a.39-a.39	a.4-d.47
a.100-c.14	a.131-a.131	a.223-d.241	a.39-a.48	a.4-d.58
a.100-c.2	a.138-a.138	a.223-d.26	a.39-b.14	a.4-d.60
a.100-c.26	a.138-d.168	a.22-a.22	a.39-b.55	a.4-d.74
a.101-a.101	a.139-b.3	a.235-b.40	a.39-b.7	a.4-d.94
a.102-a.128	a.140-a.140	a.235-d.142	a.39-b.72	a.4-e.40
a.102-b.1	a.140-b.40	a.246-c.1	a.39-c.1	a.50-b.1
a.102-b.18	a.140-c.55	a.246-d.109	a.39-d.3	a.50-j.127
a.102-b.2	a.140-d.344	a.24-a.24	a.39-d.93	a.54-g.51
a.102-b.24	a.140-g.50	a.24-a.36	a.39-g.44	a.56-d.133
a.102-b.30	a.142-a.142	a.24-b.40	a.39-g.68	a.56-d.145
a.108-d.45	a.149-d.50	a.24-c.37	a.3-a.3	a.56-d.15
a.110-d.152	a.151-d.58	a.24-c.45	a.3-b.1	a.56-d.41
a.113-c.37	a.156-b.113	a.24-d.218	a.3-b.61	a.58-c.66
a.113-c.55	a.156-d.14	a.254-a.29	a.3-b.70	a.5-a.5
a.113-d.75	a.156-g.39	a.25-g.41	a.40-a.40	a.5-c.1
a.114-c.37	a.157-d.42	a.264-a.264	a.40-c.69	a.5-c.13
a.116-d.93	a.158-b.69	a.267-a.60	a.41-d.166	a.5-d.139
a.117-a.87	a.158-c.10	a.269-c.37	a.43-a.43	a.5-d.15
a.118-a.118	a.159-d.219	a.26-a.26	a.43-d.58	a.5-d.20
a.118-a.261	a.160-d.218	a.270-c.55	a.45-c.47	a.5-d.235
a.118-a.4	a.160-d.58	a.271-d.93	a.46-c.23	a.5-d.270
a.118-a.60	a.162-a.172	a.278-d.344	a.46-c.27	a.5-d.43
a.118-a.7	a.162-c.37	a.27-c.26	a.46-d.173	a.5-d.58
a.118-b.1	a.166-b.85	a.27-d.67	a.47-b.2	a.60-a.60
a.118-b.26	a.169-b.55	a.289-a.4	a.48-c.8	a.60-b.40
a.118-b.42	a.170-b.40	a.289-b.1	a.48-d.93	a.60-c.120
a.118-b.55	a.171-b.40	a.289-c.37	a.48-g.44	a.60-c.37
a.118-b.62	a.172-c.37	a.28-a.73	a.4-a.4	a.60-c.52
a.118-b.69	a.173-d.218	a.294-a.60	a.4-a.76	a.60-c.55
a.118-b.7	a.174-c.37	a.294-b.40	a.4-a.78	a.60-d.163
a.118-b.98	a.175-d.17	a.294-c.55	a.4-b.26	a.60-d.218
a.118-c.62	a.176-c.1	a.296-c.150	a.4-b.34	a.60-d.58
a.118-c.8	a.177-a.4	a.296-d.3	a.4-b.40	a.60-e.8
a.118-c.83	a.182-d.128	a.297-b.69	a.4-b.43	a.65-a.65
a.118-c.87	a.187-a.4	a.29-a.29	a.4-b.82	a.69-a.69
a.118-d.129	a.189-a.5	a.29-c.142	a.4-b.87	a.69-c.2
a.118-d.144	a.189-d.15	a.29-d.122	a.4-c.2	a.69-c.37
a.118-d.15	a.1-a.1	a.29-d.15	a.4-c.23	a.69-d.81
a.118-d.159	a.1-b.43	a.29-e.6	a.4-c.37	a.6-b.153
a.118-d.2	a.1-c.1	a.2-a.23	a.4-c.52	a.6-d.60
a.118-d.26	a.1-c.25	a.2-a.27	a.4-c.55	a.6-g.39
a.118-d.37	a.1-c.3	a.2-b.93	a.4-c.61	a.71-c.62
a.118-d.389	a.1-d.15	a.2-d.104	a.4-c.66	a.71-d.109
a.118-d.92	a.1-d.58	a.2-d.26	a.4-c.94	a.74-a.74
a.118-e.40	a.203-d.104	a.2-d.44	a.4-d.101	a.76-b.34
a.118-f.7	a.20-b.66	a.2-g.39	a.4-d.104	a.77-a.77
a.118-g.44	a.20-d.118	a.300-c.55	a.4-d.110	a.77-c.37
a.118-g.50	a.20-d.65	a.30-b.40	a.4-d.113	a.79-c.66
a.119-b.12	a.20-d.92	a.30-d.122	a.4-d.144	a.7-a.7
a.11-b.55	a.20-g.14	a.35-a.4	a.4-d.190	a.7-d.136
a.11-d.15	a.211-c.55	a.35-b.82	a.4-d.268	a.7-d.168
a.121-a.4	a.211-d.218	a.35-d.331	a.4-d.357	a.7-d.286
a.124-b.12	a.213-d.106	a.36-c.37	a.4-d.377	a.7-d.322
				
a.80-c.37	b.14-d.3	b.1-f.14	b.38-d.136	b.55-d.15
a.83-d.128	b.150-b.71	b.1-g.16	b.38-d.79	b.59-h.1
a.85-a.86	b.150-c.1	b.1-g.41	b.3-b.3	b.60-b.60
a.85-b.1	b.153-b.40	b.1-g.94	b.3-b.30	b.61-b.61
a.86-b.1	b.153-d.229	b.1-j.128	b.3-c.1	b.61-d.3
a.86-b.112	b.160-d.335	b.1-j.131	b.3-c.56	b.61-e.3
a.87-b.55	b.160-d.7	b.22-b.22	b.40-b.40	b.61-g.12
a.89-d.58	b.163-d.169	b.23-g.3	b.40-c.37	b.61-h.1
a.8-a.8	b.169-b.169	b.24-b.30	b.40-c.66	b.62-c.1
a.8-b.130	b.179-c.1	b.24-c.37	b.40-d.104	b.62-d.74
a.8-b.30	b.179-f.60	b.26-b.26	b.40-d.122	b.64-b.64
a.8-c.6	b.181-b.181	b.29-b.29	b.40-d.142	b.66-b.66
a.91-b.55	b.181-f.4	b.29-b.42	b.40-d.15	b.66-d.92
a.91-d.144	b.18-b.18	b.29-b.67	b.40-d.169	b.66-g.14
a.92-c.30	b.18-b.23	b.29-b.68	b.40-d.230	b.68-c.51
a.92-d.142	b.18-b.3	b.29-d.166	b.40-d.51	b.68-g.12
a.93-a.93	b.18-b.30	b.29-f.1	b.40-d.52	b.68-g.3
a.93-g.3	b.18-b.6	b.29-g.75	b.40-d.58	b.69-b.69
a.96-d.113	b.18-b.68	b.29-h.4	b.40-g.45	b.69-c.69
a.96-d.129	b.18-b.69	b.2-b.2	b.41-b.43	b.69-g.16
a.97-c.26	b.18-b.77	b.2-b.3	b.41-f.23	b.6-b.6
a.98-c.7	b.18-c.1	b.2-b.42	b.42-b.42	b.6-b.69
a.99-c.28	b.18-c.23	b.2-b.6	b.42-c.1	b.6-f.17
b.103-b.85	b.18-c.41	b.2-c.1	b.42-c.68	b.70-c.69
b.103-c.57	b.18-c.69	b.2-d.126	b.42-d.281	b.71-c.1
b.105-e.3	b.18-f.1	b.2-d.166	b.42-d.3	b.71-c.23
b.106-b.106	b.18-f.8	b.2-d.93	b.43-b.43	b.72-b.72
b.106-b.40	b.19-h.3	b.2-f.1	b.43-b.44	b.72-d.26
b.107-d.58	b.1-b.1	b.30-b.71	b.43-c.20	b.73-h.1
b.108-b.40	b.1-b.120	b.30-c.1	b.43-c.23	b.76-b.85
b.108-b.68	b.1-b.18	b.30-c.2	b.43-c.25	b.77-f.1
b.108-d.2	b.1-b.2	b.30-c.6	b.43-c.26	b.78-b.78
b.109-c.1	b.1-b.29	b.30-d.17	b.43-c.37	b.7-b.7
b.109-d.157	b.1-b.3	b.30-d.82	b.43-c.85	b.7-c.1
b.110-h.4	b.1-b.30	b.31-b.31	b.43-d.14	b.7-c.10
b.113-g.39	b.1-b.40	b.33-d.129	b.43-d.15	b.7-c.19
b.117-c.37	b.1-b.61	b.34-b.34	b.43-d.58	b.7-c.45
b.117-d.242	b.1-b.69	b.34-b.40	b.43-d.67	b.7-d.144
b.118-b.118	b.1-b.7	b.34-c.37	b.44-c.37	b.7-d.15
b.11-b.11	b.1-b.71	b.34-c.44	b.44-d.250	b.80-c.1
b.121-b.121	b.1-c.1	b.34-c.55	b.45-d.333	b.80-c.102
b.122-c.1	b.1-c.10	b.34-c.56	b.46-c.65	b.80-d.153
b.122-c.116	b.1-c.17	b.34-c.66	b.47-c.62	b.80-d.92
b.122-c.26	b.1-c.23	b.34-d.104	b.47-d.170	b.81-c.68
b.122-c.66	b.1-c.69	b.34-d.144	b.47-g.18	b.82-b.82
b.122-d.17	b.1-d.105	b.34-d.177	b.48-c.55	b.82-c.100
b.122-d.265	b.1-d.144	b.34-d.211	b.49-c.37	b.82-d.220
b.12-c.69	b.1-d.159	b.34-d.3	b.4-b.4	b.84-c.30
b.130-c.55	b.1-d.169	b.34-d.315	b.50-b.50	b.84-d.142
b.131-c.62	b.1-d.176	b.34-d.93	b.52-b.7	b.85-b.85
b.132-c.13	b.1-d.19	b.34-g.41	b.52-c.37	b.85-c.1
b.133-b.80	b.1-d.272	b.34-j.129	b.52-c.81	b.85-c.57
b.141-c.132	b.1-d.3	b.36-b.36	b.52-d.31	b.92-b.92
b.142-c.52	b.1-d.353	b.36-b.47	b.53-c.26	b.92-c.1
b.149-b.18	b.1-d.58	b.36-b.68	b.55-b.55	b.98-d.92
b.149-b.71	b.1-d.92	b.37-b.37	b.55-c.1	c.104-c.72
b.149-c.1	b.1-f.10	b.38-c.55	b.55-c.45	c.108-c.37
				
c.108-c.69	c.2-c.23	c.59-c.72	d.128-d.15	d.223-d.223
c.109-c.91	c.2-c.26	c.5-c.72	d.129-d.129	d.225-d.225
c.10-c.10	c.2-c.58	c.61-c.61	d.129-d.15	d.225-d.58
c.10-d.58	c.2-d.162	c.61-d.153	d.130-d.130	d.22-g.3
c.10-g.3	c.2-d.286	c.62-d.109	d.131-d.131	d.240-e.8
c.111-c.94	c.2-d.81	c.62-g.18	d.133-d.15	d.241-g.59
c.116-d.79	c.2-e.37	c.64-d.58	d.133-d.41	d.265-d.66
c.120-e.8	c.30-c.30	c.66-d.197	d.133-d.87	d.26-d.26
c.124-c.124	c.30-d.142	c.66-d.287	d.134-d.134	d.284-d.79
c.142-d.15	c.31-c.36	c.66-d.294	d.134-d.58	d.287-d.287
c.14-c.14	c.32-d.79	c.66-g.95	d.136-d.136	d.289-d.289
c.14-c.2	c.36-c.36	c.66-j.129	d.139-d.284	d.2-e.3
c.151-c.152	c.36-c.48	c.67-d.125	d.139-d.79	d.32-d.32
c.15-c.15	c.36-c.64	c.68-d.79	d.13-d.13	d.348-d.348
c.18-d.17	c.36-d.58	c.71-c.97	d.13-d.160	d.37-d.37
c.1-c.1	c.37-c.37	c.71-d.117	d.13-d.216	d.37-f.57
c.1-c.23	c.37-c.52	c.72-c.98	d.13-d.246	d.37-g.41
c.1-c.3	c.37-c.55	c.73-d.58	d.141-d.141	d.38-d.38
c.1-c.8	c.37-c.60	c.78-c.78	d.142-d.142	d.390-j.134
c.1-d.120	c.37-d.14	c.80-d.153	d.142-g.88	d.391-d.391
c.1-d.142	c.37-d.15	c.81-d.15	d.144-d.15	d.41-d.87
c.1-d.153	c.37-d.237	c.81-d.58	d.144-d.93	d.42-d.42
c.1-d.17	c.37-d.31	c.84-c.84	d.145-d.146	d.43-d.43
c.1-d.41	c.37-d.315	c.84-d.129	d.145-d.15	d.49-d.49
c.1-d.54	c.37-d.48	c.8-c.83	d.145-d.41	d.50-d.95
c.1-d.58	c.37-d.52	c.8-d.142	d.145-d.58	d.51-d.51
c.1-d.92	c.37-d.58	c.8-d.334	d.145-d.87	d.52-d.52
c.20-c.37	c.37-e.10	c.90-e.37	d.14-d.14	d.52-d.53
c.23-c.23	c.37-f.37	c.91-c.98	d.14-d.50	d.52-f.59
c.23-c.25	c.37-g.39	c.94-c.94	d.14-d.58	d.58-d.58
c.23-c.26	c.3-c.3	c.94-d.50	d.150-d.150	d.58-d.81
c.23-c.37	c.3-c.47	c.94-d.58	d.15-d.15	d.58-f.23
c.23-c.40	c.3-d.16	c.95-c.95	d.15-d.3	d.58-g.41
c.23-c.8	c.3-d.87	c.95-d.390	d.15-d.41	d.79-d.79
c.23-d.139	c.41-d.58	c.95-j.134	d.15-d.58	d.79-d.91
c.23-d.157	c.43-c.43	c.96-d.15	d.15-d.67	d.7-d.7
c.23-d.173	c.44-c.55	c.96-d.58	d.166-d.166	d.81-h.1
c.23-d.284	c.45-c.45	c.97-c.97	d.166-d.92	d.83-d.83
c.23-d.52	c.45-d.93	d.101-d.101	d.166-f.1	d.92-h.4
c.23-d.58	c.46-c.46	d.101-d.14	d.169-d.169	d.93-d.93
c.23-d.79	c.46-d.26	d.101-d.52	d.169-f.8	d.93-g.49
c.23-e.5	c.47-c.47	d.104-d.224	d.169-g.3	d.95-d.95
c.24-c.30	c.47-d.17	d.104-d.68	d.169-h.1	d.96-d.96
c.24-c.97	c.48-c.64	d.106-d.108	d.16-d.58	e.70-f.58
c.24-d.142	c.50-c.50	d.106-d.157	d.175-e.3	f.17-f.17
c.25-d.15	c.50-c.56	d.108-d.108	d.177-d.177	f.1-h.4
c.26-c.26	c.51-d.104	d.109-d.109	d.178-d.58	f.21-f.32
c.26-c.31	c.52-c.52	d.10-d.163	d.17-d.17	f.37-f.37
c.26-c.37	c.52-d.75	d.110-d.110	d.17-d.175	f.7-f.7
c.26-d.153	c.52-d.78	d.111-g.19	d.17-g.74	g.10-g.14
c.26-d.210	c.53-c.53	d.113-d.113	d.185-d.185	g.12-g.12
c.26-d.229	c.55-c.55	d.113-g.41	d.193-g.81	g.12-g.3
c.26-d.308	c.55-d.127	d.114-d.159	d.198-d.198	g.14-g.14
c.26-d.52	c.55-d.75	d.11-d.11	d.211-d.211	g.14-g.27
c.26-d.66	c.55-d.79	d.11-e.3	d.211-g.45	g.14-g.3
c.27-d.41	c.55-e.8	d.120-d.176	d.218-d.58	g.14-g.32
c.2-c.2	c.56-d.58	d.122-d.14	d.21-d.21	g.16-g.16
				
g.18-g.18	g.27-g.3	g.39-g.39	g.41-g.41	g.7-g.7
g.23-g.3	g.37-g.37	g.3-g.3	g.51-g.51	g.89-g.93
g.24-g.24	g.37-g.44	g.3-g.32	g.66-g.66	g.8-g.8
g.27-g.27	g.38-h.1	g.3-g.68	g.69-g.69	g.9-g.9

**Table 2:** Number of occurrences of folds that interact only with protein domains of same fold solely in SCOPe database

Fold	Number of occurrences
b.118	3
a.142	4
d.289	4
a.131	6
b.169	6
g.66	6
d.49	10
d.391	13
d.83	13
d.348	14
a.264	16
b.31	16
a.101	18
b.4	19
b.37	20
g.69	25
b.64	58
d.223	77
c.15	79
b.78	81
a.65	88
d.198	115
g.9	120
d.21	139
g.24	139
d.150	152
b.11	157
d.130	179
d.141	179
c.53	191
c.43	209
g.7	269
g.8	277
d.185	310
a.26	325
b.22	337
c.124	346
d.131	463
a.126	488
d.32	498
a.74	543
d.96	636
c.78	679
a.22	707
d.38	883
b.60	976
b.121	1504
b.50	2681

**Table 3:** Folds that associate with 20 or more number of folds and the number of functions associated with these folds (860 fold-fold combinations are observed

Name of the fold	SCOP ID of the fold	Number of fold-fold combinations	Number of functions associated with the fold
Ribonuclease H-like motif fold	c.55	20	7
Flavodoxin-like fold	c.23	23	10
Beta-Grasp (ubiquitin-like) fold	d.15	27	12
OB-fold	b.40	28	9
Alpha-alpha superhelix fold	a.118	31	20
TIM beta⁄alpha-barrel fold	c.1	36	16
Ferredoxin-like fold	d.58	37	25
DNA/RNA-binding 3-helical bundle fold	a.4	39	6
P-loop containing nucleoside triphosphate hydrolases	c.37	42	1
Immunoglobulin-like beta-sandwich fold	b.1	43	14
